# Ameliorative Effect of *Moringa oleifera* Against CUMS-Induced Anxiety in Rats: β-Catenin and 5-HT_1 A_ Crosstalk

**DOI:** 10.1007/s12035-025-04911-8

**Published:** 2025-04-23

**Authors:** Rana A. El-Kadi, Mohamed S. Sedeek, Noha F. Abdelkader, Hala F. Zaki, Ahmed S. Kamel

**Affiliations:** 1 Alexandria University Hospitals, Champollion Street, El-Khartoum Square, El Azareeta, Alexandria City, 21131 Egypt; 2 Pharmacognosy and Medicinal Plants Department, Faculty of Pharmacy, Cairo University, Kasr El-Aini, Cairo City, 11562 Egypt; 3 Pharmacognosy Department, Faculty of Pharmacy, King Salman International University, Ras-Sedr, South Sinai City, 46612 Egypt; 4 Pharmacology and Toxicology Department, Faculty of Pharmacy, Cairo University, Kasr El-Aini, Cairo City, 11562 Egypt

**Keywords:** *Moringa oleifera*, Anxiety, 5-HT_1 A_, β-Catenin, Serotonin

## Abstract

Serotonin 1 A receptor (5-HT1 AR) signaling is pivotal for stress response, determining vulnerability or resilience to psychopathology. However, the precise pathological mechanisms underlying its role remain inconsistent. *Moringa oleifera* (MO), a plant with purported medicinal properties, has demonstrated potential efficacy against psychiatric disorders. However, no available information exists regarding its effects on 5-HT1 A signaling under normal and stressed conditions. This study is aimed at elucidating the effects of MO in conjunction with 5-HT1 A signaling. Rats were randomly assigned to four groups: normal (NRML), normal rats receiving MO orally at 200 mg/kg (MO), rats exposed to chronic unpredictable mild stress (CUMS) for 21 days (CUMS), and stressed rats administered MO from day 15 (CUMS + MO). Behavioral analysis was conducted using forced swimming and open field tests. Serotonergic markers, β-catenin, p-Erk, c-myc, and mTOR were assessed via ELISA, while miRNA clusters and individual miRNAs were analyzed using PCR. No significant differences were observed between the NRML and MO groups, both of which exhibited approximately normal biochemical activity, except for a decreased 5-HIAA/5-HT ratio in the MO group, which was reflected behaviorally. Rats subjected to CUMS displayed defective β-catenin signaling, potentially leading to compensatory activation of 5-HT1 A. Consistently, the CUMS + MO group exhibited normalized 5-HT1 A and 5-HT signaling, accompanied by reduced pThr183-Erk and its downstream targets, c-myc and miR- 203, to mitigate pathological anxiety. Additionally, mTOR and its downstream target, miR- 217, were reduced compared to stressed rats. MO exhibited a promising anxiolytic effect by modulating 5-HT1 A signaling, as evidenced by improved neurobehavioral outcomes and restoring biochemical balance in stressed rats. These findings highlight its potential therapeutic role in anxiety management.

**Graphical Abstract:**

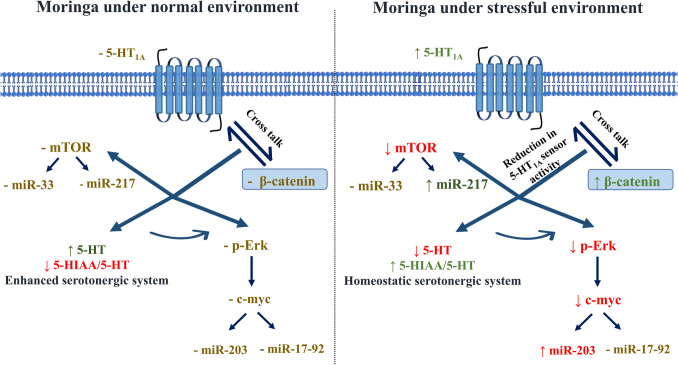

## Introduction

Stress is a significant contributing factor to various diseases, including stroke, heart attack, and psychiatric disorders [1]. Psychiatric disorders account for 13% of the global disease burden, with anxiety being the most prevalent [2]. Anxiety is an adaptive emotional response to harmful stimuli; however, when excessive and persistent, it leads to pathological anxiety [3]. Moreover, anxiety accounts for 26,800,000 disability-adjusted life years (DALYs) worldwide [4, 5]. Unfortunately, anxiety is a costly condition and is considered a chronic disease that does not resolve spontaneously.

Stress can disrupt normal brain physiology, leading to psychopathology, although some individuals exhibit resilience and effectively cope with it [6]. Consequently, understanding the neurological mechanisms underlying susceptibility or resilience to chronic unpredictable mild stress (CUMS)-induced disorders is essential for elucidating the pathophysiology of these debilitating diseases, which remains largely obscure. Serotonin (5-HT) is a key mediator in the central nervous system that modulates behavior and brain activity [7]. Notably, stimulation of hippocampal serotonergic neurotransmission may facilitate adaptation to severe, inescapable stressors [8]. Moreover, serotonergic metabolism in the hippocampus is higher in female rats than in males, reflecting the nearly twofold higher prevalence of anxiety disorders in females [9]. Serotonin is primarily metabolized into 5-hydroxy- 3-indolacetic acid (5-HIAA) [9]. Agonists of the serotonin 1 A receptor (5-HT1 AR) have been found to decrease both 5-HT and 5-HIAA levels [8].

The 5-HT1 AR is a key target within the serotonergic system, regulating the release of 5-HT and other neurotransmitters [10]. It governs both adaptive and maladaptive responses, significantly influencing social behavior following stress exposure [11]. As a well-established target in psychotropic therapy, 5-HT1 AR plays a crucial role in mood regulation, stress resilience, and cognitive function [12]. Furthermore, stress-induced behavioral abnormalities in the hippocampus can be mitigated by 5-HT1 AR activation [13]. Interestingly, both agonists and antagonists of 5-HT1 AR have demonstrated antidepressant effects in preclinical studies. However, the role of 5-HT1 AR antagonists in anxiety remains debated, as they have exhibited both anxiogenic and anxiolytic effects in mice [14, 15].

Serotonin receptors exert diverse, context-dependent effects on cognition, influenced by factors such as drug dosage and receptor regulation [15]. Evidence suggests that high doses of 5-HT1 AR agonists impair memory, whereas low doses enhance cognitive function, indicating a dose-dependent effect [15]. Moreover, hippocampal serotonergic activation modulates basolateral amygdala (BLA)-dependent long-term potentiation (LTP), particularly under stress conditions [16]. Notably, post-training intra-BLA microinjection of the 5-HT1 AR antagonist (S)-WAY- 100135 reverses stress-induced memory impairment, further supporting its differential role under stressful conditions. Additionally, stress-induced state-dependent learning is mediated via BLA 5-HT1 AR [17].

Beyond 5-HT1 AR, other serotonin receptor subtypes exhibit bidirectional effects on cognition. Recent findings indicate that 5-HT3 receptor antagonists, such as tropisetron and granisetron, enhance neuronal count in the CA1 hippocampal region [18]. Furthermore, 5-HT4 receptor activation promotes LTP, whereas 5-HT6 receptor stimulation reduces LTP, impairing learning [15]. These findings highlight the dynamic and context-dependent nature of serotonin receptor signaling. However, understanding of 5-HT1 AR activation, transduction mechanisms, and associated effects remains limited, particularly regarding its paradoxical relationship with anxiety. These alterations in 5-HT transmission offer a novel perspective on brain plasticity and may provide insights into the neurobiology of anxiety-related disorders.

Indeed, 5-HT1 AR signaling and stress regulation are closely linked to extracellular signal-regulated kinases (Erk), microRNAs (miRNAs), and β-catenin. Stress can suppress Erk signaling [19], whereas increased β-catenin levels and resilience-associated miRNAs can alleviate depression [20]. Stressed rats exhibit significantly reduced hippocampal ERK1/2 and diminished ERK1/2-driven phosphorylation of downstream targets, such as ribosomal S6 kinase 1 and mitogen- and stress-activated kinase 1. These key effectors play crucial roles in stress resilience, and their dysfunction has been implicated in neuropsychiatric disorders [21]. However, conflicting findings exist regarding the activation of Erk signaling in the hippocampus by 5-HT1 AR [22].

Conversely, miRNAs serve as critical regulators of resilience and anxiety, playing a fundamental role in the pathophysiology of mental disorders. This highlights miRNA dysregulation as a significant factor in affective disorders and underscores their potential as biomarkers or therapeutic targets [23]. Based on genomic organization, miRNAs are classified into clusters and individual miRNAs. Moreover, miRNA clusters consist of multiple miRNAs transcribed from a single primary transcript, regulating proliferation, differentiation, and survival. One such cluster, miR- 17–92, is pivotal for hippocampal neurogenesis and influences anxiety [24]. Individual miRNAs exert more specific regulatory effects, potentially targeting distinct aspects of disease pathology. Some single miRNAs modulate glucocorticoid signaling pathways and exhibit antidepressant effects, likely through regulating 5-HT1 AR and the serotonin transporter (SERT) [25].

β-Catenin, a structural protein that regulates approximately 400 genes, is thought to be modulated by 5-HT1 AR, leading to either stimulation or inhibition of cellular machinery [26, 27]. Notably, Dias et al. (2014) demonstrated that β-catenin overexpression in mice exerted pro-resilient, anxiolytic, and antidepressant-like effects [28]. Thus, β-catenin is considered a critical regulator of behavioral resilience, activating a network that includes Dicer1 and downstream miRNAs. Accordingly, modulation of Erk, miR- 17–92, and β-catenin represents a crucial therapeutic target for stress-related disorders.

One of the traditional herbal medicines that has garnered significant scientific attention is *Moringa oleifera* (MO). It is highly valued for its abundance of vitamins, minerals, antioxidants, and the growth hormone zeatin, with its leaves being the most extensively studied for their therapeutic potential [29]. The therapeutic effects of MO leaves have been demonstrated in various disorders, including chronic inflammation, hypertension, immunological disorders, hyperglycemia, and hyperlipidemia [30]. Moreover, MO has been widely investigated for its anxiolytic potential, with studies indicating its therapeutic application in anxiety management [31]. Notably, nanoparticles derived from MO leaf extract have been shown to exert anxiolytic effects by enhancing serotonergic signaling, suggesting a mechanistic link to mood regulation [32]. Furthermore, MO has demonstrated antidepressant-like properties attributed to its ability to modulate the noradrenergic-serotonergic neurotransmission pathway, a mechanism similar to that of selective serotonin reuptake inhibitors [33]. Although MO has been shown to enhance serum 5-HT levels and modulate hippocampal neuronal activity [34], mechanistic insights underlying its anxiolytic effects remain unclear, necessitating further investigation [35].

Beyond its role in neurotransmitter modulation, MO exhibits bidirectional regulation of the Erk signaling pathway, suggesting a context-dependent effect on neuronal plasticity and stress responses [36, 37]. Additionally, MO influences β-catenin signaling, a critical neurodevelopment and synaptic function regulator, further supporting its potential role in neuropsychiatric disorders [38, 39]. MO contains conserved and novel miRNAs in its leaves, seeds, and calluses under both stressed and unstressed conditions, which may contribute to its regulatory effects on gene expression and neural homeostasis [40]. Ultimately, the anxiolytic properties of MO through 5-HT1 AR signaling and its downstream targets, including Erk, miRNAs, and β-catenin, require further elucidation.

This study evaluated the efficacy of MO in alleviating anxiety in adolescent models, focusing on its modulation of 5-HT1 AR signaling. The precise role of hippocampal 5-HT1 AR in psychiatric disorders remains unclear, particularly regarding adaptive responses to stress. Given the urgent need to delineate how 5-HT1 AR influences susceptibility and resilience to anxiety, this study is aimed at elucidating MO’s impact on hippocampal 5-HT1 AR signaling in both normal and CUMS-exposed female rats.

## Materials and Methods

### Animals

Female Wistar Albino rats, adolescent (6 weeks old), weighing 150–180 g, were obtained from the animal facility of Faculty of Pharmacy, Cairo University (Cairo, Egypt). Rats were kept in optimal housing circumstances, 60% ± 10% humidity level, and a room temperature of 25 ± 2 °C. The animals received a standard laboratory food and water ad libitum. All procedures were carried out in compliance with the “Research Ethical Committee” of Faculty of Pharmacy, Cairo University (Approval Number: PT (2548)) and the National Institutes of Health guide for the care and use of laboratory animals (NIH publication No. 85–23, revised 2011).

### Drugs and Chemicals

Aqueous extract of MO leaves was purchased from the National Research Center, Cairo, Egypt. One milliliter concentrated MO aqueous extract contains 1 g MO grinded leaves.

### Experimental Protocol

**Fig. 1:**
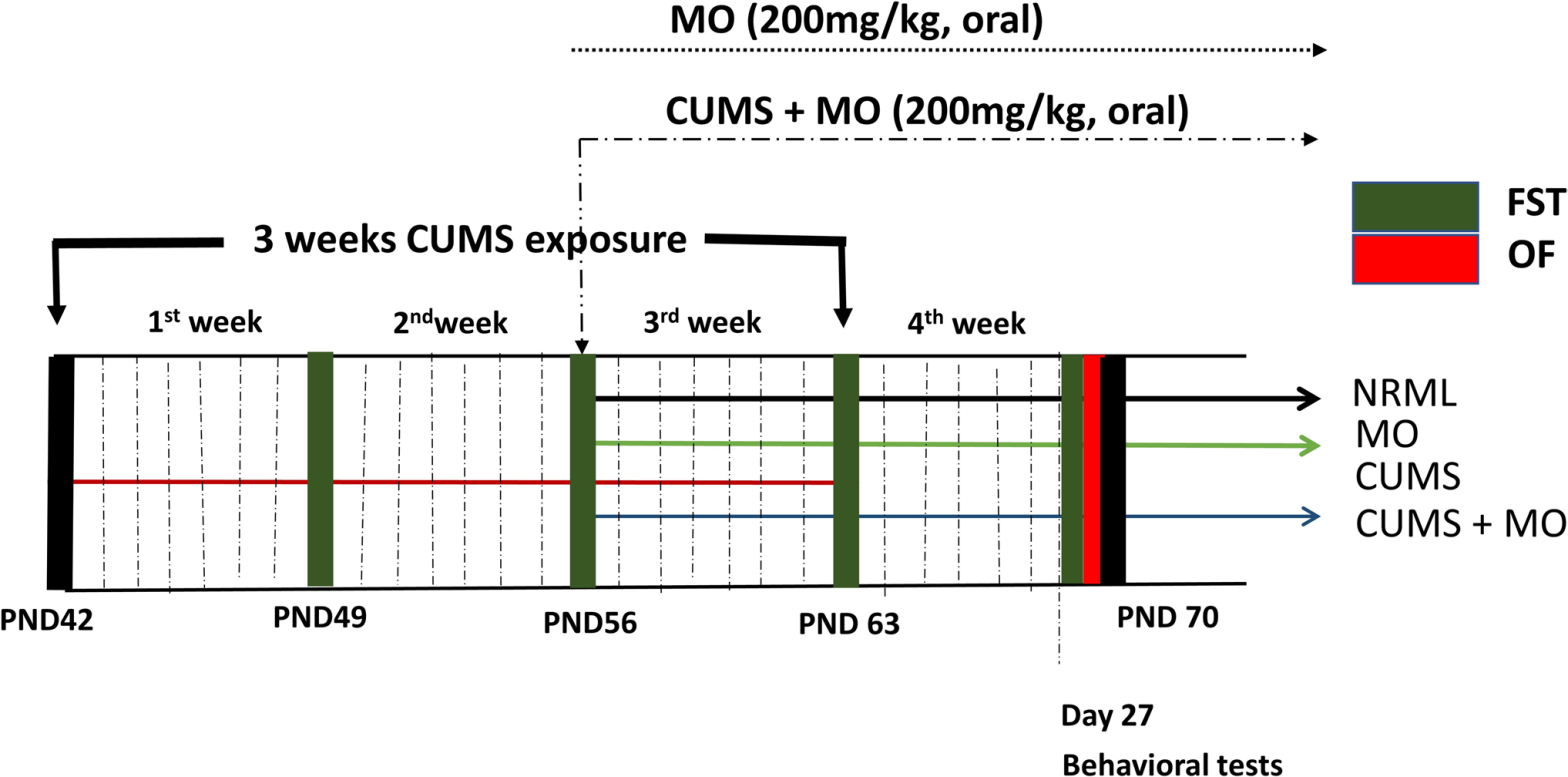
Experimental design for the study

**Fig. 2:**
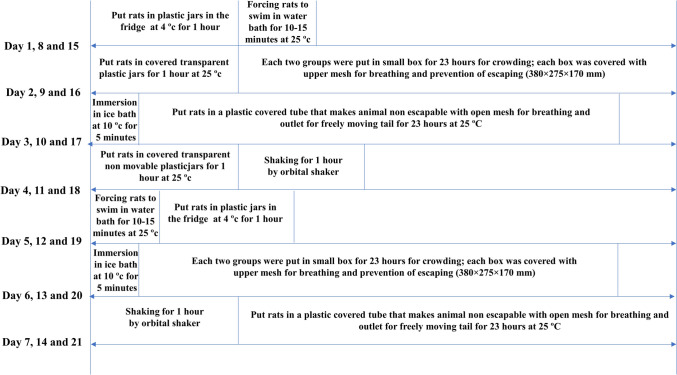
Time schedule for CUMS protocol

The experimental design is represented in Fig. 1. CUMS was carried out by exposing rats to various stresses for 21 days (Fig. 2) [41]. Forty rats were randomly assigned to four experimental groups (ten rats in each group). The sample size was calculated using prior literature on CUMS as a reference and the G-Power software (version 3.1, Düsseldorf, Germany) with an effect size = 0.6, an alpha level = 0.05, a statistical power = 0.8, and number of groups = 4. Group I served as control group (NRML) and received distilled water. Group II (MO) received MO (200 mg/kg, oral) [35] for 14 days starting from day 15. Group III (CUMS) was subjected to CUMS protocol and received distilled water. Group IV (CUMS + MO) was subjected to CUMS then treated with MO. Depression was evaluated by forced swimming test (FST) at days 7, 14, 21 and 27 for NRML and CUMS groups, while anxiety was assessed by open field test (OFT) at day 27 for all groups. Animals were euthanized by cervical dislocation under isoflurane anesthesia [42].

### CUMS Protocol

The CUMS protocol was applied for 21 consecutive days to induce anxiety- and depression-like behaviors in animals. The stressors were randomly scheduled to prevent habituation and ensure unpredictability. Animals were exposed to two stressors per day. Cold immobilization and FST were performed at days 1, 5, 8, 12, 15, and 19. Immobilization and crowding were applied at days 2, 4, 9, 11, 16, and 18. Forced cold swimming was applied at day 3, 6, 10, 13, 17, and 20, while isolation was carried out at days 3, 7, 10, 14, 17, and 21. Vibration was applied at days 4, 7, 11, 14, 18, and 21. The procedure was shown in Fig. 2 [41, 43].

### Forced Swimming Test

The FST was carried out to evaluate depression state where immobility reflected behavioral despair of rats. Rats were individually placed in a transparent Plexiglas cylinder (50 cm height × 20 cm diameter) filled with water at a depth of 15 cm (maintained at 23–25 °C) and allowed to swim freely for 6 min [44]. This depth ensured the swimming of rats and prevented their contact with the cylinder’s base for support. The animals were trained 1 day before the testing day. For each rat, the total immobility time was recorded within the standardized 5-min test period. The total immobility time was the period when the rat ceased swimming and remained floating on the water’s surface, with minimal effort keeping its head above water. To prevent the animals’ hypothermia, body temperature was maintained under a heating lamp [20].

### Open Field Test

The OFT was performed to evaluate rodents’ exploratory actions and general activity. The test was carried out using a square wooden box (80 × 80 × 40 cm). Each rat was gently placed in the center of the open field and was allowed to freely explore the area for 5 min; then, the floor was cleaned after each tested animal. Rat behaviors were recorded using a video camera placed to the top of the box; then, the ANY-Maze video tracking software (Stoelting Co., Wood Dale, Illinois, USA) was used for behavioral analysis. Total distance traveled, mean speed, time immobile, and central/thigmotaxis time were recorded. Positioning of the animal in the periphery indicates anxiety. However, its tendency toward the center indicates anti-stress action. Hence, central/thigmotaxis time ratio is expressed as the anxiety index, where time spend in central zone is compared to lateral one [45].

### Brain Processing

Rats were decapitated under anesthesia 1 week following CUMS (day 28). The brains of animals were gathered, washed, dried, and weighed. Hippocampi were dissected and flash frozen in liquid nitrogen. After that, the specimens were stored at − 80 °C. The hippocampi were split into two groups. The first group (*n* = 5) was used for enzyme-linked immunosorbent assay (ELISA) analysis of 5-HT_1 A_R, 5-HT, 5-HIAA, β-catenin, p-Erk, c-myc, and mTOR. The other one (*n* = 5) was for quantitative real-time-PCR (qRT-PCR) analysis of miR- 17–92, miR- 203, miR- 33, and miR- 217.

### Enzyme-Linked Immunosorbent Assays

Rat-specific enzyme-linked immunosorbent assay kits were used according to the manufacturer’s instructions. The parameters that were determined; 5-HT_1 A_R (MyBiosource, San Diego, USA, Cat. No.: MBS928226), 5-HT (MyBiosource, San Diego, USA, Cat. No.: MBS2700308), 5-HIAA (MyBiosource, San Diego, USA, Cat. No.: MBS024867), mTOR (MyBiosource, San Diego, USA, Cat. No.: MBS744326), β-catenin (MyBiosource, San Diego, USA, Cat. No. MBS843456), p-Erk (Thr183) (MyBiosource, San Diego, USA, Cat. No. MBS267200), and c-Myc (MyBiosource, San Diego, USA, Cat. No. MBS2 511134). The levels were expressed as picogram per milligram for 5-HT_1 A_-R and nanogram per milligram for 5-HT, 5-HIAA, and mTOR.

### Quantitative Real-Time-PCR

Following homogenization of brain tissues, total RNA was extracted using the Mirvana kit (Thermo Fisher, USA, Cat. No.: A27828) according to the manufacturer’s instructions. The produced miRNA was quantified using a Nanodrop® spectrophotometer at 260 nm. The TaqMan® MicroRNA Assays are intended to identify and quantify mature micRNAs utilizing Applied Biosystems real-time PCR devices. cDNA is reverse transcribed from miRNA samples in the reverse transcription (RT) stage using particular miRNA primers from the TaqMan® MicroRNA Assays and chemicals from the TaqMan® MicroRNA Reverse Transcription Kit (Thermo Fisher, USA, Cat. No.: 4366596). Each 15 µL RT reaction contains 7 µL of master mix, 3 µL of primer, and 5 µL of miRNA sample. The master mix composed of 1 µL MultiScribe™ Reverse Transcriptase (Thermo Fisher, USA, Cat No: 4311235), 4.16 µL nuclease-free water, 50 U/µL, 1.50 µL 10× reverse transcription buffer, 0.15 µL 100 mM dNTPs (with dTTP), and 0.19 µL RNase inhibitor 20 U/µL. Requirements for the amplification step comprised 10 min at 95 °C for promotion of AmpliTaq Gold DNA polymerase. Afterward, denature stage with 40 cycles at 95 °C for 15 s after that annealing/extension phase at 60 °C for 1 min. Eventually, the expression of the chosen gene was normalized in reference to the mean critical threshold (CT) of the values of miR (U 6) housekeeping gene expression using the ΔΔCt method [46]. Primer sequence for miR- 17–92, miR- 203, miR- 33, and miR- 217 genes is displayed in Table 1.

**Table 1:** The primer sequence of the miR- 17–92, miR- 203, miR- 33, and miR- 217 genes

Gene symbol	Primer sequence
miR- 17–92	Forward (F): 3′-CAGTAAAGGTAAGGAGAGCTCAATCTG- 5′ Reverse (R): 3′-CATACAACCACTAAGCTAAAGAATAATCTGA- 5′
miR- 203	Forward (F): 5′-GTA TCC AGT GCA GGG TCCGA- 3′ Reverse (R): 5′-CGA CGG TGA AAT GTT TAG- 3′
miR- 33	Forward (F): 5′-GGCACTACTTCTGATCCTTC- 3′ Reverse (R): 5′-CAACTACAATGCACCACAGCTG- 3′
miR- 217	Forward (F): 5′-CACGTGCAGCCGTTTAAGCCGCGT- 3′ Reverse (R): 5′-TTCCCATTCTAAACAACACCCTGAA- 3′
miR (U 6) House keeping	Forward (F): 5′- CTCGCTTCGGCAGCACA- 3′ Reverse (R): 5′-AACGCTTCACGAATTTGCGT- 3′

### Determination of Total Phenolic Content

The total phenolic content was carried out using the Folin–Ciocalteu method according to the procedure described previously [47]. A stock solution of gallic acid (2 mg/mL in methanol) was prepared, and then different dilutions were prepared: 750, 500, 375, and 250 μg/mL. Ten microliters of sample/standard was mixed with 100 μL of Folin-Ciocalteu reagent (diluted 1: 10) in a 96-well microplate. Then, 80 μL of 1 M Na2 CO3 was added and incubated in the dark at room temperature for 20 min. At the end of incubation time, the absorbance of the resulting blue color was measured at 630 nm. The results were recorded using a microplate reader FluoStar Omega.

### Determination of Total Flavonoid Content

The total flavonoid content was carried out using the aluminum chloride method [48], with minor modifications to be carried out in microplates. A stock solution of standard rutin (2000 μg/mL in methanol) was prepared; then, various dilutions were prepared: 500, 250, 125, 62.5, 31.25, and 15.625 μg/mL. Briefly, 15 μL of sample/standard was placed in a 96-well microplate; then, 175 μL of methanol was added followed by 30 μL of 1.25% AlCl3. Finally, 30 μL of 0.125 M C2H3 NaO2 was added and incubated for 5 min. At the end of incubation, the absorbance of the resulting yellow color was measured at 420 nm using a microplate reader FluoStar Omega.

### Determination of Total Tannin Content

The total tannin content was determined using ferric chloride method according to a previous procedure [49], with minor modifications to be carried out in microplates. A stock solution of standard tannic acid was prepared at 500 μg/mL in distilled water; then, various dilutions were prepared: 62.5, 31.25, 15.625, 7.8125, and 3.90625 μg/mL. Briefly, 20 μL of 1% FeCl3 was placed followed by 20 μL of 1% K4[Fe(CN)6] in a 96-well microplate. Finally, the volume was completed to 200 μL with distilled water, and the mixture was incubated for 5 min. At the end of incubation, the absorbance of the resulting yellow color was measured at 720 nm. The results were recorded using a microplate reader FluoStar Omega.

### Statistical Analysis

All obtained data were reported as mean ± S.D. The results were examined using one-way ANOVA, followed by Tukey’s multiple comparison tests. When normality failed, non-parametric one-way ANOVAs (Kruskal–Wallis one-way ANOVA on ranks, followed by Dunn’s post hoc test) were done, and when homogeneity of variance failed, Welch’s ANOVA and differences between two groups were analyzed using the Games-Howell test. Statistical analysis was carried out using SPSS software (version 26) (IBM Corp., Armonk, New York). The threshold of significance was set at *p* 0.05 for all statistical tests, and graph pad prism software was used to present the graphical design (version 8) (San Diego, CA, USA). *F*-value (F), degrees of freedom (df), and statistical significance (*p*) were reported.

## Results

### *Moringaoleifera* Mitigates Anxiety-Like Behavior in Stressed Rats

**Fig. 3:**
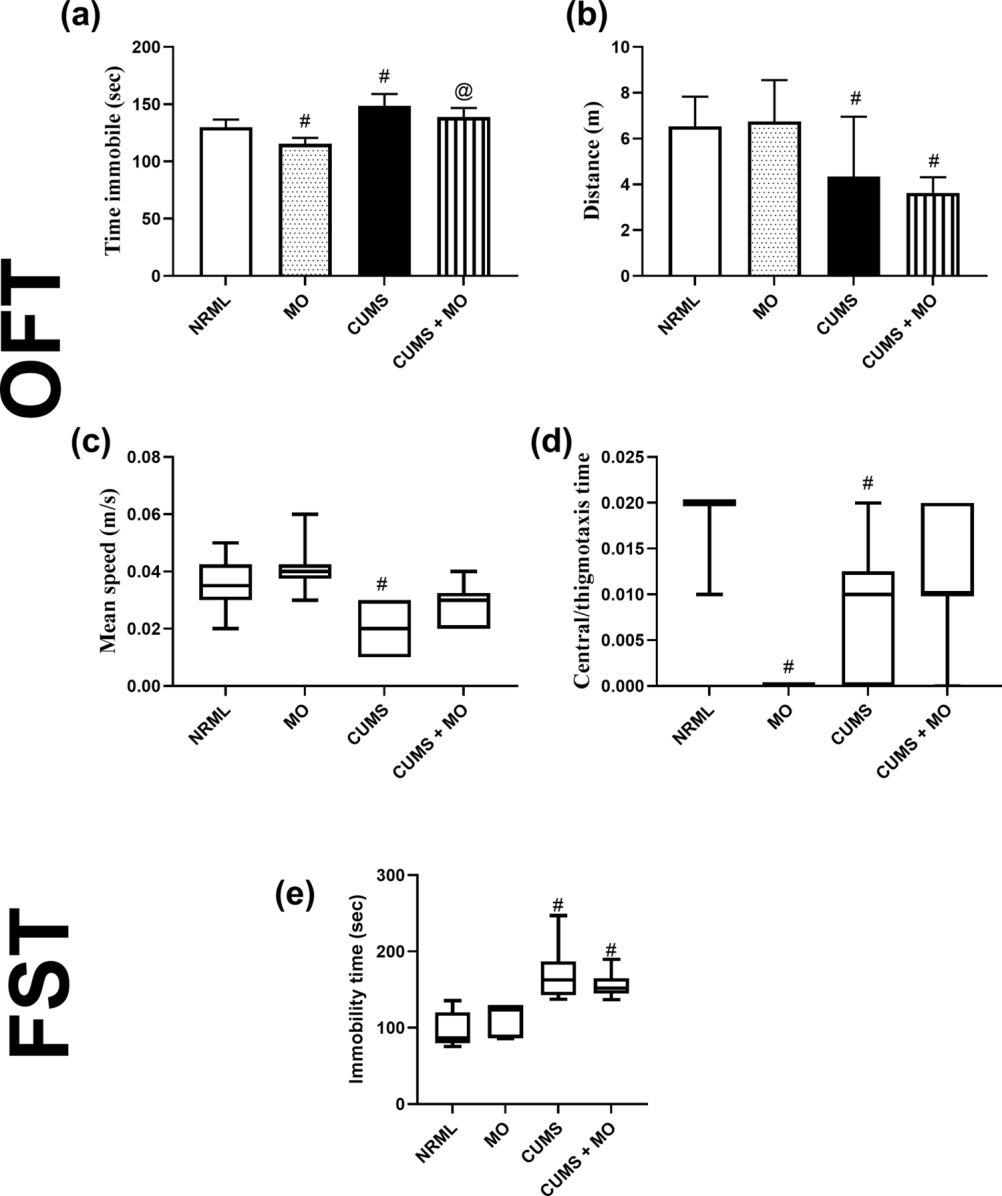
Effect of MO on anxiety-like behavior in OFT and the first week of depression in FST. Panels represent **a** time immobile, **b** distance, **c** mean speed, **d** central/thigmotaxis time, and **e** immobility time. Each bar with vertical line represents mean ± S.D. of ten rats per group. Statistics: **a** one-way ANOVA followed by Tukey’s post hoc test; **b** one-way ANOVA followed by Games-Howell’s post hoc test; **c–e** Kruskal–Wallis one-way ANOVA followed by Dunn’s post hoc test; ^#^ vs. NRML and ^@^ vs. CUMS

The FST and OFT tests are estimators for depression and anxiety, respectively. The results of OFT were presented in Fig. 3a–d. Rats with CUMS showed elevated immobility time (*F*_(3.36)_ = 32.61; *P* < 0.001) by 14.3% and decreased total distance (*F*_(3.36)_ = 7.95; *P* < 0.001) by 33.6%. The CUMS group exhibited a significant reduction in mean speed (0.02 vs. 0.035 in NRML, *P* < 0.001) and central/thigmotaxis time (0.01 vs. 0.02 in NRML, *P* < 0.001), reflecting absolute decreases of 0.015 and 0.01, respectively. Conversely, MO-treated rats displayed no significant differences in total distance and mean speed (*P* > 0.05) compared to the NRML group, with 11.1% of immobility time. However, MO-treated rats completely eliminated central/thigmotaxis time (0.00 vs. 0.02 in NRML, *P* < 0.001), reflecting a 100% absolute reduction.

In the CUMS + MO group, immobility time was normalized (no significant difference vs. NRML, *P* > 0.05), as were mean speed (0.03 vs. 0.035 in NRML, *P* > 0.05) and central/thigmotaxis time (0.01 vs. 0.02 in NRML, *P* > 0.05). However, total distance remained reduced, showing 44.4% and 16.3% decrease compared to NRML and CUMS, respectively (*P* < 0.05).

In the FST (Fig. 3e), depressive-like behavior was observed in week 1, where CUMS (162.6 vs. 86.7 in NRML, *P* < 0.001) and CUMS + MO (151.8 vs. 86.7 in NRML, *P* < 0.001) exhibited significantly higher immobility times, with absolute increases of 75.9 and 65.1, respectively. However, this depressive-like behavior disappeared from the second week onward (data not shown).

### *Moringaoleifera* Enhances Hippocampal Weight and Balances β-Catenin and p-Erk in Stressed and Normal Rats

**Fig. 4:**
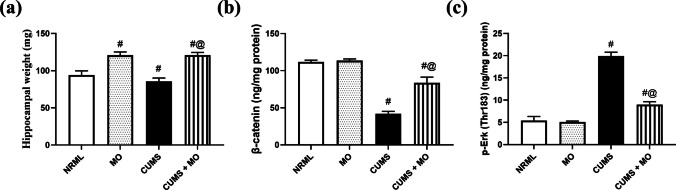
Effect of MO on **a** hippocampal weight, **b** β-catenin, and **c** p-Erk in stressed rats. Results are expressed as the mean ± SD. Each bar with vertical line represents mean ± S.D. (*n* = 6 in hippocampal weight and *n* = 5 in biochemical analysis). Statistics: **a, c** one-way ANOVA followed by Tukey’s post hoc test; **b** one-way ANOVA followed by Games-Howell’s post hoc test; ^#^ vs. NRML and ^@^ vs. CUMS

In Fig. 4, CUMS decreased hippocampal weight (*F*_(3.20)_ = 93.13; *P* < 0.001) by 8.9% and β-catenin signal (*F*_(3.16)_ = 294.96; *P* < 0.001) by 62.4% compared to NRML group. Furthermore, CUMS increased p-Erk signal (*F*_(3.16)_ = 435.35; *P* < 0.001) by 2.7-fold in comparison with NRML group. The group receiving MO exhibited normal β-catenin and p-Erk signals with a 28.5% elevation in hippocampal weight relative to NRML group. Rats of CUMS + MO group exhibited an increase in β-catenin expression and hippocampal weight by 99% and 41.4%, respectively, along with a decrease in p-Erk by 54.7% in comparison with CUMS group.

### *Moringaoleifera* Modulates Hippocampal Serotonergic System

**Fig. 5:**
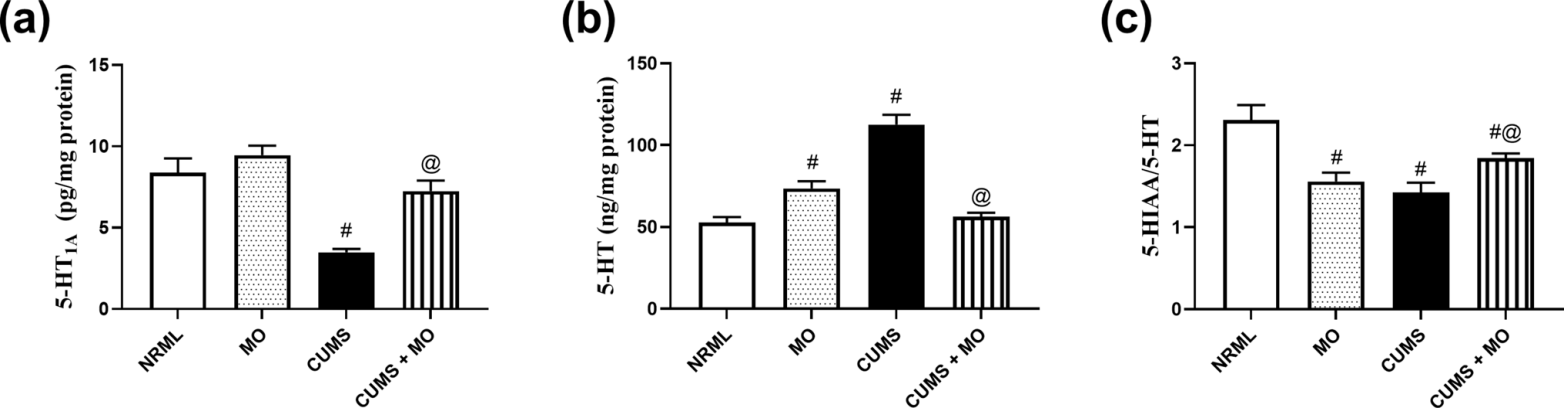
Effect of MO on **a** 5-HT_1 A_R,_,_ **b** 5-HT, and **c** 5-HIAA/5-HT in stressed rats. Results are expressed as the mean ± SD. Each bar with vertical line represents mean ± S.D. of five rats per group. Statistical analysis was performed using one-way ANOVA followed by Tukey’s post hoc test, ^#^ vs. NRML and ^@^ vs. CUMS

In Fig. 5, CUMS decreased 5-HT_1 A_ (*F*_(3.16)_ = 78.90; *P* < 0.001) and 5-HIAA/5-HT ratio (*F*_(3.16)_ = 48.52; *P* < 0.001) by 58.5% and 38.2%, respectively, and increased 5-HT (*F*_(3.16)_ = 207.57; *P* < 0.001) by 1.13-fold as compared to NRML group. Rats of MO group exhibited normal 5-HT_1 A_; however, MO increased 5-HT by 39.6% and reduced 5-HIAA/5-HT ratio by 32.4% compared to NRML group. CUMS + MO group reverted 5-HT_1 A_ and 5-HT to normal values and increased 5-HIAA/5-HT ratio by 29.2% compared to CUMS group.

### *Moringaoleifera* Modulates p-Erk Downstream Targets

**Fig. 6:**
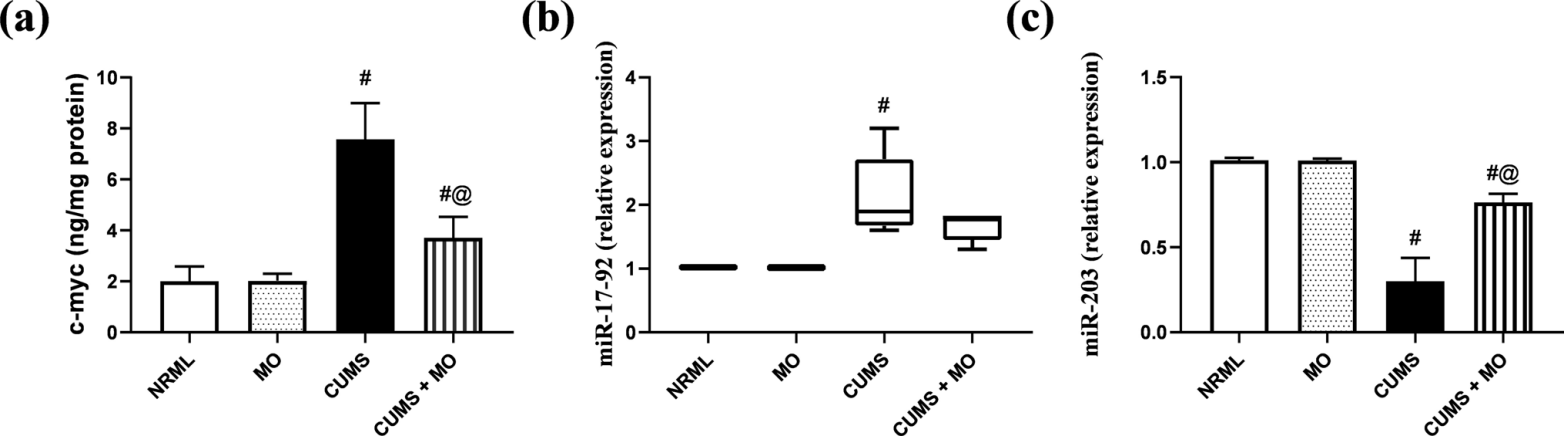
Effect of MO on **a** c-myc, **b** miR- 17–92, and **c** miR- 203 in stressed rats. Results are expressed as the mean ± SD of 5 rats per group. Statistics: **a** one-way ANOVA followed by Tukey’s post hoc test; **b** Kruskal–Wallis one-way ANOVA followed by Dunn’s post hoc test; **c** one-way ANOVA followed by Games-Howell’s post hoc test; ^#^ vs. NRML and ^@^ vs. CUMS

In Fig. 6, CUMS significantly raised c-myc (*F*_(3.16)_ = 44.47; *P* < 0.001) by 2.8-fold, increased miR- 17–92 (1.9 vs. 1.02 in NRML, *P* < 0.01), and decreased miR- 203 (*F*_(3.16)_ = 103.13; *P* < 0.001) approximately by 70%-fold compared to NRML. MO group exhibited normal c-myc, miR- 17–92 (1.01 vs. 1.02 in NRML), and miR- 203 signals relative to NRML group. CUMS + MO normalized miR- 17–92 (1.8 vs. 1.9 in CUMS), decreased c-myc signal by 51%, and increased miR- 203 signal by 1.6-fold compared to CUMS group.

### *Moringaoleifera* Modulates mTOR with Its Downstream Targets

**Fig. 7:**
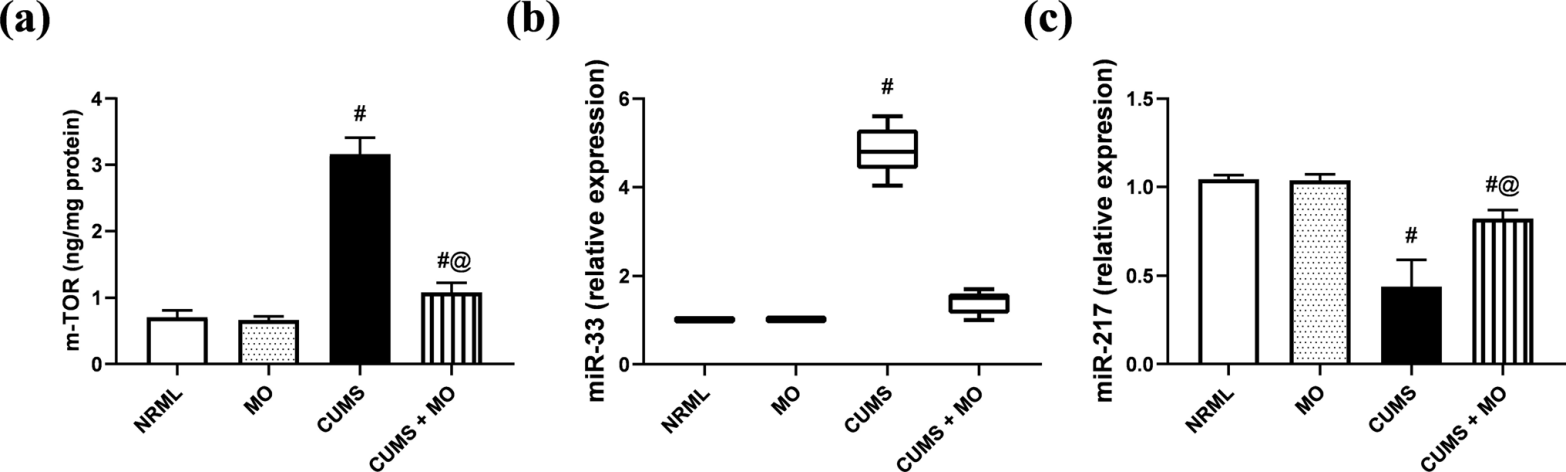
Effect of MO on **a** mTOR, **b** miR- 33, and **c** miR- 217 in stressed rats. Results are expressed as the mean ± SD of 5 rats per group. Statistics: **a, c** one-way ANOVA followed by Tukey’s post hoc test; **b** Kruskal–Wallis one-way ANOVA followed by Dunn’s post hoc test, ^#^ vs. NRML and ^@^ vs. CUMS

In Fig. 7, CUMS increased mTOR (*F*_(3.16)_ = 289.42; *P* < 0.001) by 3.5-fold and miR- 33 (4.8 vs. 1.01 in NRML, *P* < 0.01), while decreased miR- 217 (*F*_(3.16)_ = 59.5; *P* < 0.001) by 58% in comparison with NRML group. The MO group exhibited normal mTOR, miR- 33 (1.01 vs. 1.01 in NRML), and miR- 217. CUMS + MO normalized miR- 33 (1.5 vs. 4.8 in CUMS), decreased mTOR by 66%, and increased miR- 217 signal by 88% in comparison with CUMS group.

### Determination of Total Phenolic, Flavonoid, and Tannin Contents

**Fig. 8:**
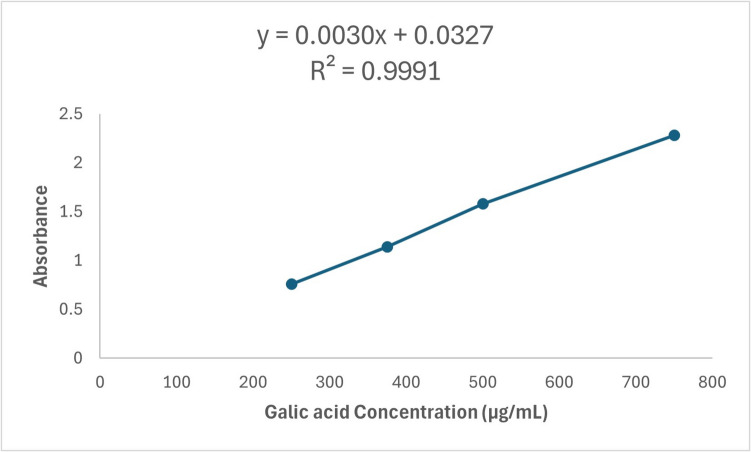
Standard calibration curve of gallic acid

**Fig. 9:**
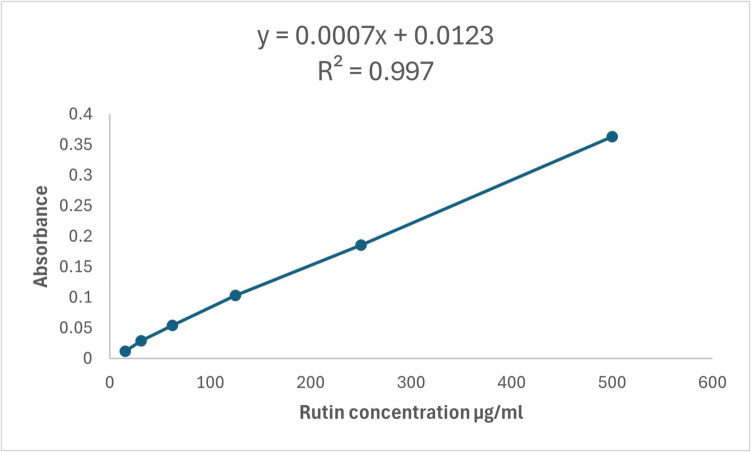
Standard calibration curve of rutin

**Fig. 10:**
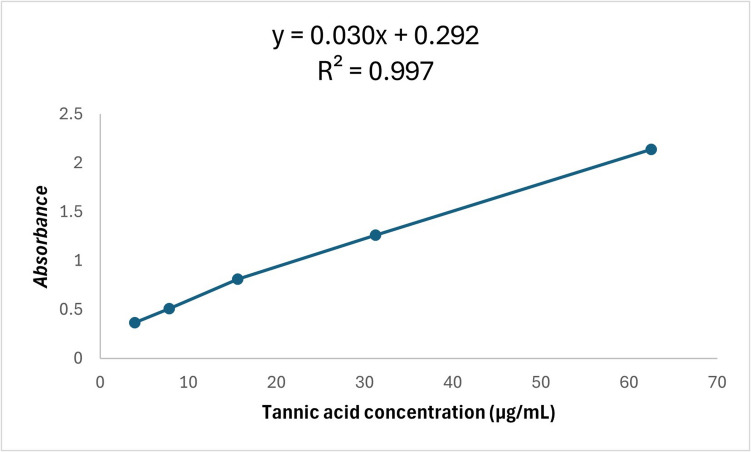
Standard calibration curve of tannic acid

In Fig. 8, the total phenolic content of MO aqueous extract was estimated as gallic acid equivalent (Table 2). Using the gallic acid standard as demonstrated in Fig. 8. The studied MO leaves aqueous extract showed a considerable total phenolics amount of 273.99 ± 16.28 μg/mL GAE. In Fig. 9, flavonoids are one of the major pharmacologically active phenolic constituents of MO leaves extract. It is estimated as rutin equivalent (Table 3) using rutin standard as illustrated in Fig. 9, and the total flavonoid content of the studied extract is 82.43 ± 1.01 μg/mL rutin equivalent. In Fig. 10, the MO leaf extract is rich in numerous phenolic compounds, which are considered the major effectors of its pharmacological activities [50]. Tannin content in the studied MO leaves was determined as illustrated in Table 4 and Fig. 10 as a tannic acid equivalent. Total tannins in the extract were 207.58 ± 9.06 μg/mL TAE, which is considered a reasonable amount.

**Table 2:** Total phenolic content of MO leaf aqueous extract

Sample	Absorbance (630 nm)	Total phenolic content (μg gallic acid/1 mL sample)	Standard deviation
MO leaf aqueous extract	0.83	273.99	16.28

**Table 3:** Total flavonoids content of MO leaf aqueous extract

Sample	Absorbance at (420 nm)	Total flavonoid content (μg rutin/1 mL sample)	Standard deviation
MO leaf aqueous extract	0.06	82.43	1.01

**Table 4:** Total tannin content of MO leaf aqueous extract

Sample	Absorbance (720 nm)	Total tannin content (μg tannic acid/1 mL sample)	Standard deviation
MO leaf aqueous extract	0.86	207.58	9.06

## Discussion

To the best of our knowledge, the results of the current study provide the first evidence for the role of 5-HT1 AR signaling in MO’s effects under both normal and stressed conditions. The anxiolytic effect of MO under stressful conditions may be mediated by the crosstalk between 5-HT1 AR and β-catenin signaling. Multiple lines of evidence support this: (i) reduction in anxious behavior manifestations; (ii) elevation of β-catenin signaling; (iii) normalization of 5-HT1 AR and 5-HT signaling; (iv) increase in the 5-HIAA/5-HT ratio; (v) attenuation of exaggerated p-Erk, c-myc, and mTOR levels; and (vi) upregulation of miR- 203 and miR- 217.

The current study utilized adolescent rats, as adolescence is characterized by ongoing hippocampal maturation, heightened neuroplasticity, and increased vulnerability to stress-related disorders [51]. Six-week-old rats are highly susceptible to anxiety and extinction deficits, making them an appropriate model for investigating MO's effects on 5-HT1 AR signaling [52]. This age group is translationally relevant to early adolescence in humans, a critical period of susceptibility to anxiety-related disorders that often persist into adulthood [53]. Early-life stress is known to induce permanent epigenetic and structural changes in the hippocampus, increasing the risk of chronic anxiety disorders [54–57]. Given the limited availability of effective pharmacological treatments for adolescent anxiety, novel interventions such as MO are urgently needed [58–61].

The OFT was utilized to assess anxiety-like behavior [62]. Untreated CUMS rats exhibited anxiety, as indicated by increased immobility time and decreased total distance traveled, mean speed, and central/thigmotaxis time. Following MO treatment, behavioral improvements were observed in all parameters except total distance traveled, which remained unchanged compared to CUMS rats. Similar findings have been reported with anxiolytic drugs such as diazepam and mitragynine, where the total distance traveled was not significantly altered [63]. Notably, total distance traveled is not a definitive measure of anxiety, as supported by Bunck et al. (2009) [64], and may reflect stress-coping mechanisms rather than hyperactivity, as suggested by Zangrandi et al. (2021) [65]. The decrease in central/thigmotaxis time following MO treatment may be attributed to elevated 5-HT levels or may represent a normal anxiety response, as reported by Muigg et al. (2009) and Singewald et al. (2007) [66, 67].

The observed increase in hippocampal weight in the MO group may be attributed to its neuroprotective and neurotrophic effects, potentially mediated through BDNF modulation, enhancement of neurogenesis, and synaptic remodeling during adolescence. The hippocampus undergoes significant neurodevelopmental changes during this period, characterized by heightened neurogenesis, synaptic remodeling, and increased volume [68, 69]. Given the high plasticity of the adolescent brain, external influences, including environmental and neurochemical factors, can modulate hippocampal development and function [70, 71]. Disruptions in hippocampal neurogenesis during adolescence have been linked to long-term stress-related consequences [69]. Moreover, BDNF, a key neurotrophic factor, plays a crucial role in neuronal survival, synaptic plasticity, and memory [72].

Additionally, inflammation induced by a Western diet has been shown to reduce hippocampal weight and disrupt the blood–brain barrier, though the precise mechanisms remain unclear [72, 73]. The current findings align with previous studies highlighting the responsiveness of the adolescent hippocampus to neurochemical influences [70, 71, 74]. However, given the limited research on hippocampal changes during adolescence, further studies are necessary to elucidate MO’s potential role in promoting hippocampal health during this critical neurodevelopmental phase [75].

Animal studies have revealed that hippocampal serotonergic activity through 5-HT1 AR can influence susceptibility or resilience to stress [76]. Through its receptor, 5-HT promotes neurogenesis and counteracts depression, thereby facilitating adaptive strategies. Conversely, it exerts an anxiogenic effect [77, 78]. Moreover, the 5-HT metabolite 5-HIAA determines the neurological state of the brain and serves as a measure of serotonergic activity. The 5-HIAA/5-HT ratio is influenced by the type of stress exposure [79]. It has been shown that reducing hippocampal Erk levels and modulating serotonergic signaling by lowering 5-HT levels and increasing the 5-HIAA/5-HT ratio in the prefrontal cortex can alleviate anxious behaviors in mice. This effect is associated with reduced blood plasma corticosterone levels, a key marker of stress reduction [80]. Li et al. (2022) suggested that a low 5-HIAA/5-HT ratio compensates for deficient 5-HT1 AR signaling [81].

Additionally, Van Praag (1994) linked aggressive and anxiogenic behaviors to reduced 5-HIAA levels [82], as observed in the CUMS group in the present study. This finding supports the anxiolytic effects of MO in the CUMS + MO group, where 5-HT levels normalized, and the 5-HIAA/5-HT ratio increased, ensuring efficient serotonergic neurotransmission in the presence of reduced 5-HT1 AR signaling in the CUMS group [81].

Herein, MO enhanced mood under normal conditions by boosting serotonergic activity, increasing 5-HT levels, and reducing its metabolite 5-HIAA, a pattern associated with resilience and well-being [83]. However, under CUMS, MO modulated the serotonergic system differently, reducing 5-HT levels, increasing 5-HT metabolism, and downregulating 5-HT1 AR and β-catenin, thereby mitigating the exaggerated compensatory response. This study is the first to highlight MO’s dual regulatory role in serotonergic modulation under normal and stressed conditions. Additionally, a decreased 5-HIAA/5-HT ratio, an indicator of antidepressant effects, was observed in the CUMS group [84]. Notably, none of the rats exhibited sustained depressive symptoms, as reflected in reduced immobility time in the FST beyond the first week.

Furthermore, hippocampal resilience may be influenced by the crosstalk between β-catenin and 5-HT1 AR signaling [85]. In the CUMS group, 5-HT1 AR, β-catenin, and hippocampal weight were reduced, potentially triggering a compensatory mechanism involving 5-HT, Erk, and mTOR signaling. The downregulation of 5-HT1 AR and β-catenin may contribute to anxiety development, as supported by previous studies [86, 87]. However, MO treatment under CUMS conditions normalized 5-HT1 AR expression and enhanced β-catenin signaling, reinforcing its anxiolytic effects. Reduced hippocampal 5-HT1 AR expression has been implicated in anxiety, consistent with findings by Zhu et al. (2020) [88], although the precise mechanism remains unclear [89]. The interplay between 5-HT1 AR and β-catenin may underlie anxiety pathophysiology, as their upregulation mitigates exaggerated 5-HT, p-Erk, and c-myc signaling, consistent with Xiang et al. (2018), further supporting MO’s anxiolytic effects under stress [90]. These findings align with the core conclusions of the present study.

Erk plays a dual role in stress adaptation, exhibiting both antidepressant and anxiogenic properties [91]. Like 5-HT signaling, Erk’s adaptive mechanisms depend on hippocampal 5-HT1 AR activity [92]. Studies suggest that 5-HT1 AR can activate Erk directly or through serotonergic signaling, as previously highlighted [93, 94]. However, the signal transduction of 5-HT1 AR remains controversial. Gharib et al. (2018) associated low hippocampal 5-HT1 AR levels with reduced Erk activity, whereas Xiang et al. (2018) reported increased hippocampal Erk levels despite reduced 5-HT1 AR expression [95, 96]. In vivo findings suggest that 5-HT1 AR can suppress hippocampal Erk activity [81]. Reduced 5-HT1 AR signaling may lead to compensatory Erk activation or increased serotonergic activity, as described by Li et al. (2022) [97]. Furthermore, modulation of serotonergic signaling and hippocampal Erk inhibition, as seen with ylang-ylang oil, which increases the 5-HIAA/5-HT ratio in the prefrontal cortex, has been shown to reverse anxiety-like behaviors [80].

The hippocampal neurogenic role of Erk has been well documented [97], suggesting that the downregulation of 5-HT1 AR may induce an adaptive response through Erk-driven neurogenesis, potentially providing antidepressant effects at the expense of increased anxiety, as described by Quaglia et al. (2017). This is consistent with findings from Marin et al. (2011), where 5-HT1 AR knockout (KO) mice exhibited heightened anxiety despite displaying antidepressant activity in the FST [98]. These observations suggest that 5-HT1 AR modulates Erk signaling either directly or via 5-HT, contributing to stress resilience, which aligns with the findings of this study. Furthermore, the 5-HT1 AR agonist 8-OH DPAT has been shown to enhance 5-HT1 AR signaling while suppressing dorsal hippocampal 5-HT firing, producing an anxiolytic effect [99]. Similarly, Chang et al. (2020) proposed that anxiolysis may result from increased 5-HT1 AR signaling coupled with decreased Erk activity, a mechanism closely resembling MO’s role in alleviating psychiatric anxiety under CUMS conditions [100].

Beyond 5-HT1 AR-Erk interactions, c-myc, a key downstream effector of Erk, plays a significant role in hippocampal neurogenesis [101, 102] and regulates miRNA synthesis, including miR- 203 and miR- 17–92, which are involved in anxiety regulation [24, 103, 104]. In CUMS-exposed rats, MO treatment significantly reduced Erk and c-myc expression while increasing miR- 203 levels compared to untreated CUMS rats. The suppression of Erk and its downstream target c-myc in CUMS + MO rats aligns with prior studies by Edwinanto et al. (2018) and Pappas et al. (2021) [105, 106]. MO had no significant effects on these pathways under normal conditions, suggesting that its regulatory effects are specifically activated in response to stress-induced alterations. MO restored miR- 17–92 expression and normalized Erk, c-myc, and miR- 203 signaling exclusively under stress, indicating a homeostatic mechanism. The increase in miR- 203 may be attributed to the suppression of Erk and c-myc, further supported by the absence of MO’s effect on these pathways under normal conditions.

Additionally, the elevated c-myc levels observed in the CUMS group align with findings from Xiang et al. (2018), which identified Erk and c-myc as key contributors to anxiety-related behaviors [90]. Erk and mTOR are downstream targets of 5-HT1 AR, implicated in antidepressant effects [107]. Furthermore, mTOR regulates miR- 33 and miR- 217, both of which play crucial roles in cell viability, differentiation, and neurogenesis [108–111]. Under stressed conditions (CUMS), MO inhibited mTOR while increasing miR- 217, aligning with its isothiocyanate-mediated mTOR suppression, which has been linked to anti-tumor activity with minimal toxicity [112]. Conversely, MO did not significantly affect mTOR, Erk, or their downstream targets under normal conditions, suggesting a stress-specific regulatory mechanism.

Additionally, miR- 17–92 and miR- 33 were elevated under stress, yet MO did not significantly affect their expression in normal or stressed conditions. Notably, loss of miR- 17–92 in knockout (cKO) mice induced depressive-like behaviors, including increased immobility in the FST and TST and reduced sucrose consumption, whereas miR- 17–92 overexpression exhibited antidepressant effects [113]. In the present study, CUMS-exposed rats displayed elevated miR- 17–92 levels, potentially explaining the absence of depressive-like behavior in this group. However, while miR- 17–92 deletion had no impact on hippocampal weight in mice, the CUMS group exhibited reduced hippocampal weight, suggesting that further research is warranted to explore the role of miR- 17–92 in hippocampal integrity in rats.

This study prioritized the neuropharmacological evaluation of MO, particularly its impact on 5-HT1 A receptor signaling in anxiety models based on the previous established phytochemical profile of Egyptian MO leaves, as reported by El-Sohaimy et al. (2015) [114] and Abo El-Fadl et al. (2020) [115]. As previously mentioned, the studied MO leaf aqueous extract exhibited a considerable total phenolic content. Phenolic compounds in MO leaves are considered the primary mediators of their health benefits [50, 116]. MO leaves and their isolated phenolic constituents have demonstrated potent antioxidant, anti-inflammatory, and anticancer activities [117, 118]. The aqueous extract of MO leaves contains various biologically active phenolic metabolites that significantly reduce oxidative stress, protect against neurodegenerative disorders, and improve behavioral abnormalities, including anxiety and depressive behaviors [119, 120]. The flavonoid content of the studied MO leaf aqueous extract was estimated.

Flavonoid components of MO leaf extract, such as kaempferol and quercetin, have been reported as potent neuropharmacological active compounds, contributing to neuroprotective and anti-neuroinflammatory activities [120]. These flavonoids have demonstrated significant anxiolytic and antidepressant properties [121]. Tannin, one of the major phenolic constituents of MO leaves, is present at the highest concentration in this plant [122, 123]. Tannin-rich plant extracts have potent anxiolytic effects [124], suggesting that the high tannin content in MO leaves is a key contributor to its anxiolytic activity.

While the present study confirms the presence of total phenolics, flavonoids, and tannins, it does not focus on isolating and characterizing individual compounds or their specific interactions with signaling pathways and 5-HT receptor activity. The authors acknowledge this limitation and emphasize that future studies should employ advanced chromatographic techniques, bioassays, and receptor-binding studies to identify the key bioactive metabolites responsible for MO’s anxiolytic effects. Furthermore, many studies used total phenolics, flavonoids, and tannins as quantitative indicators of active constituents, correlating them with the biological activity of medicinal plant extracts [125, 126]. This approach is widely accepted for initial pharmacological assessments before further chemical characterization. A limitation of this study is the absence of fingerprint analyses, which could have provided a detailed profile of MO extract and allowed for correlation between specific bioactive compounds and the observed anxiolytic effects. Such analyses could further elucidate the precise phytochemical constituents responsible for MO’s neuropharmacological activity.

## Conclusion

Based on environmental conditions, MO can modulate its downstream targets. Additionally, the crosstalk between 5-HT1 AR and β-catenin alters neuronal excitability through Erk, 5-HT, and mTOR signaling, providing protective effects against depression while potentially exacerbating anxiety.

## Data Availability

The data supporting this study’s findings are available from the corresponding author upon reasonable request.

## Abbreviations

5-HIAA: 5-Hydroxyindoleacetic acid
5-HT: Serotonin
5-HT_1 A_R: Serotonin 1 A receptor
BLA: Basolateral amygdala
BSA: Bovine serum albumin
CUMS: Chronic unpredictable mild stress
DALYs: Disability adjusted life years
DG: Dentate gyrus
ELISA: Enzyme-linked immunosorbent assay
FST: Forced swimming test
LTP: Long-term potentiation
Mir- 203: MicroRNA 203
MiRNAs: MicroRNAs
MO: *Moringa oleifera*
mTOR: Mammalian target of rapamycin
OFT: Open field test
p-Erk: Phosphorylated; extracellular signal-regulated kinase
PCR: Polymerase chain reaction
qRT-PCR: Quantitative real-time-PCR
SDS-PAGE: Sodium dodecyl sulfate polyacrylamide gel electrophoresis
